# Platypnoea–orthodeoxia syndrome affects nocturnal oxygen desaturation: A case report

**DOI:** 10.1002/rcr2.1106

**Published:** 2023-02-16

**Authors:** Mamiko Hoshi, Yutaka Kozu, Michio Kawamura, Naho Furusho, Ryosuke Ozoe, Yusuke Jinno, Kenichi Sugaya, Hisato Hiranuma, Kazuo Chin, Yasuhiro Gon

**Affiliations:** ^1^ Division of Respiratory Medicine Nihon University School of Medicine Tokyo Japan

**Keywords:** atrial septal defect, platypnoea–orthodeoxia syndrome, sleep apnoea syndrome

## Abstract

A woman in her late 20s who had received an implantable cardioverter defibrillator in childhood for ventricular arrhythmia was diagnosed with severe obstructive sleep apnoea (apnoea–hypopnoea index, 77.1/h), and she began continuous positive airway pressure treatment. Before initiating this treatment, she had moderate hypoxaemia of unknown cause. She was admitted for adjustment of the position of her implantable cardioverter defibrillator, which had caused purple discoloration and ulceration of the overlying skin. On admission, she had dyspnoea and her arterial oxygen saturation by pulse oximetry significantly decreased while sitting. This led to detection of a patent foramen ovale and a right‐to‐left shunt while sitting. We diagnosed platypnoea–orthodeoxia syndrome with an atrial septal defect. Atrial septal defects should be suspected in hypoxic patients with obstructive sleep apnoea.

## Graphical abstract

We herein describe a patient with severe sleep apnoea who was diagnosed with platypnoea–orthodeoxia syndrome (POS) after identification of unexplained hypoxia. The changes in SpO2 before and after surgery were characteristic of POS.

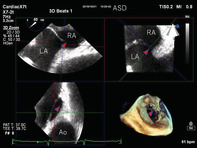

## INTRODUCTION

Platypnoea–orthodeoxia syndrome (POS) is characterized by arterial desaturation while in an upright or sitting position. Its causes are categorized as cardiac and noncardiac. Cardiac causes have anatomic and functional components, whereas noncardiac causes include ventilation/perfusion mismatch and pulmonary shunts. Anatomic abnormalities frequently associated with POS include intracardiac shunts caused by an atrial septal defect (ASD) or patent foramen ovale (PFO). The presence of a functional element that increases blood flow through the cardiac shunt and elevates the right‐to‐left pressure gradient, such as pulmonary hypertension or a misplaced atrial septum, results in hypoxaemia. Although the percutaneous oxygen saturation (SpO_2_) changes with the body position, changes during sleep have rarely been reported. POS is difficult to diagnose in patients with an ASD or PFO. We herein describe a patient with severe sleep apnoea who was diagnosed with POS after identification of unexplained hypoxia. The changes in SpO_2_ before and after surgery were characteristic of POS.

## CASE REPORT

A woman in her late 20 s (body mass index, 21.6 kg/m^2^) had received an implantable cardioverter defibrillator (ICD) for ventricular arrhythmia in childhood. One year before admission, she was diagnosed with severe obstructive sleep apnoea (OSA) [apnoea–hypopnoea index (AHI), 77.1/h; apnoea index, 11.5/h; hypopnoea index, 62.9/h] and began continuous positive airway pressure (CPAP) treatment. Polysomnography data 1 year before admission were as follows: total sleep time, 272 min; awake time, 43.9%; non‐rapid eye movement (NREM) sleep Stage 1, 14.8%; Stage 2, 18.1%; Stage 3, 17.6%; rapid eye movement (REM) sleep, 5.6%; AHI, 74.3/h; central apnoea index, 0.2/h; obstructive apnoea index, 11.3/h; hypopnoea index, 62.9/h; respiratory disturbance index for total sleep time, 74.3/h (REM, 102.5/h; NREM, 71.2/h); mean SpO_2_, 94%; lowest SpO_2_, 80%; cumulative percentage of sleep time with SpO_2_ of <90% and <85% (CT90 and CT85, respectively), 20.9% and 0.6%, respectively. Auto‐adjusting (4–12 cmH_2_O) CPAP treatment was started (DreamStation; Philips, Amsterdam, the Netherlands). According to the CPAP tracking system, the AHI was maintained at <10/h.

**FIGURE 1:**
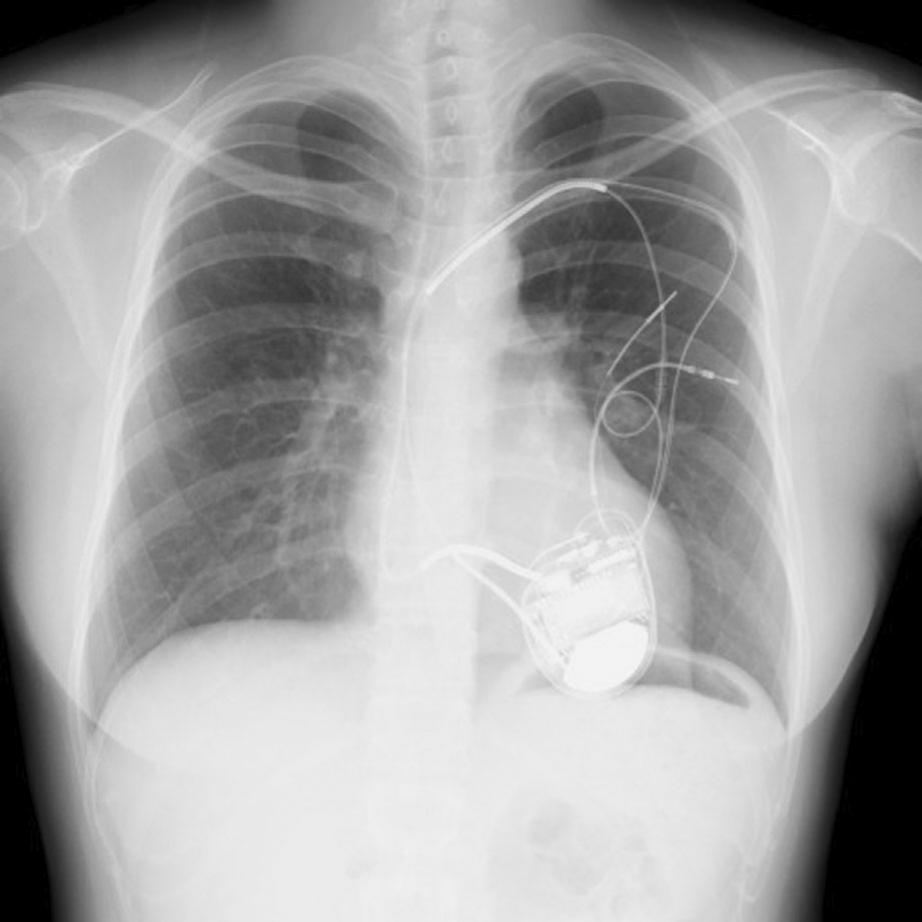
Chest radiograph. No pulmonary abnormalities were present

**FIGURE 2:**
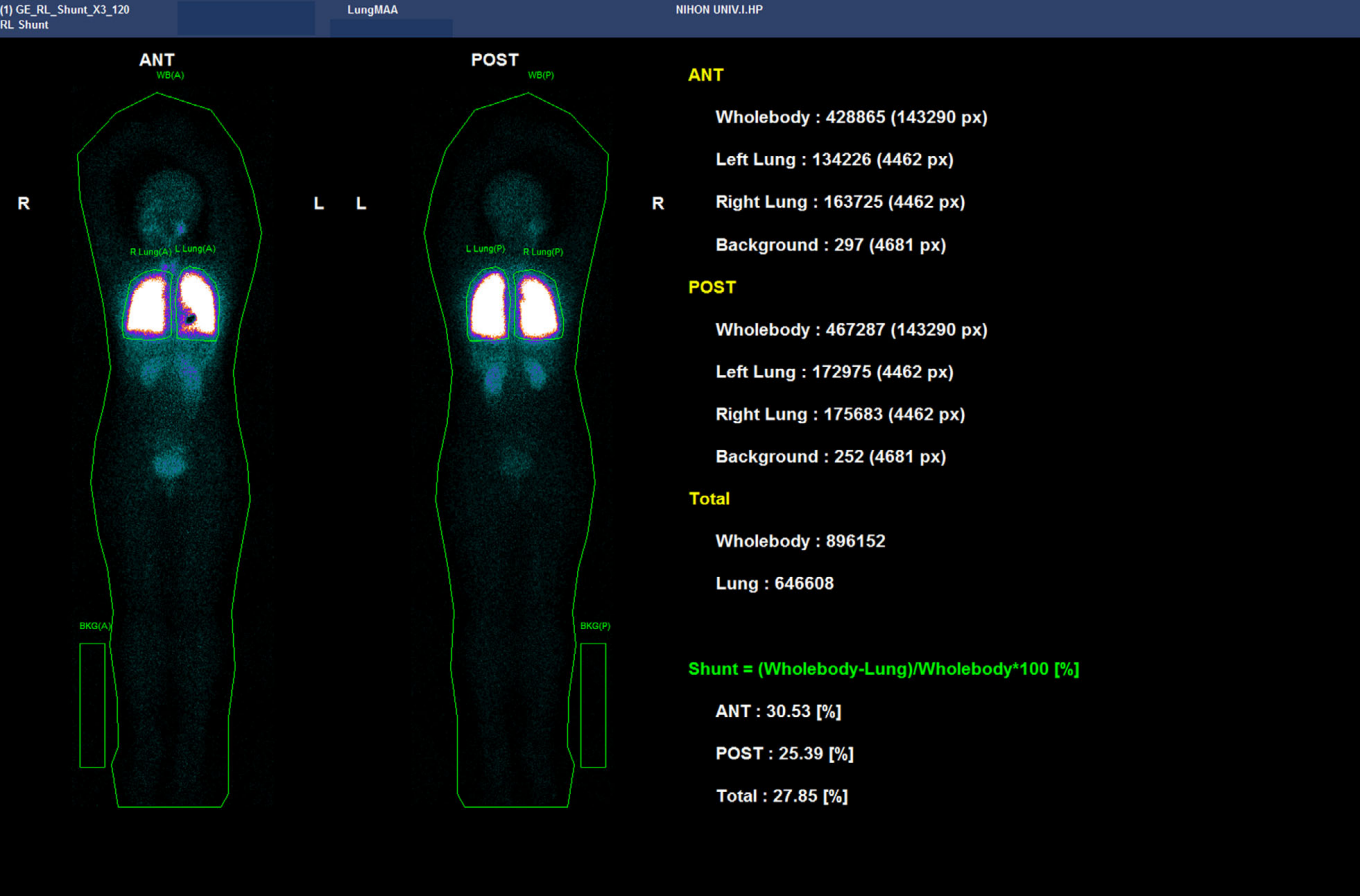
Lung perfusion scintigraphy. Uptake in the kidneys suggested an intrapulmonary right‐to‐left shunt. Shunt rate: 27.9%

**FIGURE 3:**
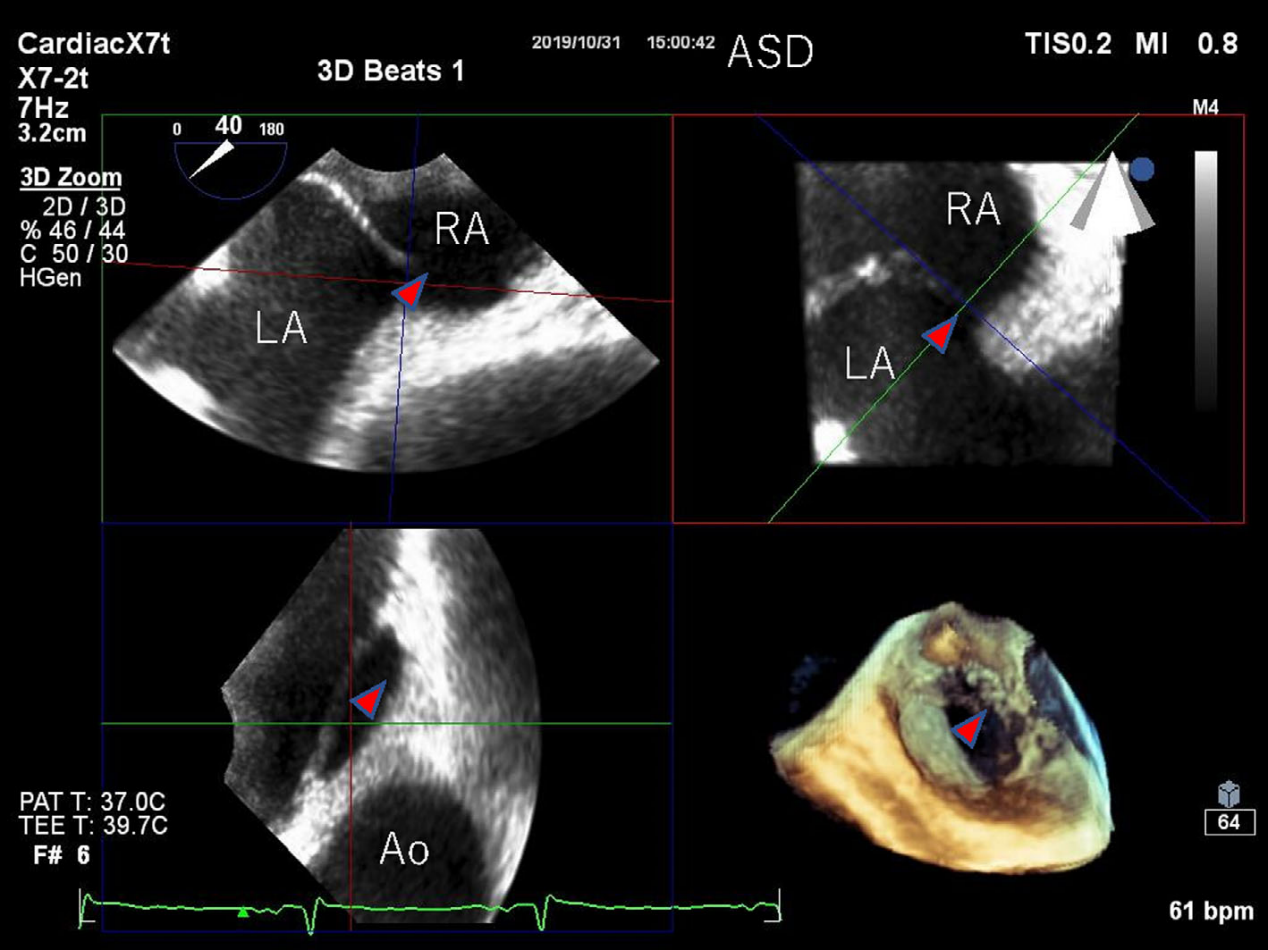
Trans‐oesophageal echocardiography in the sitting position. Ao, aorta; ASD, atrial septal defect (red arrow); LA, left atrium; RA, right atrium

**FIGURE 4:**
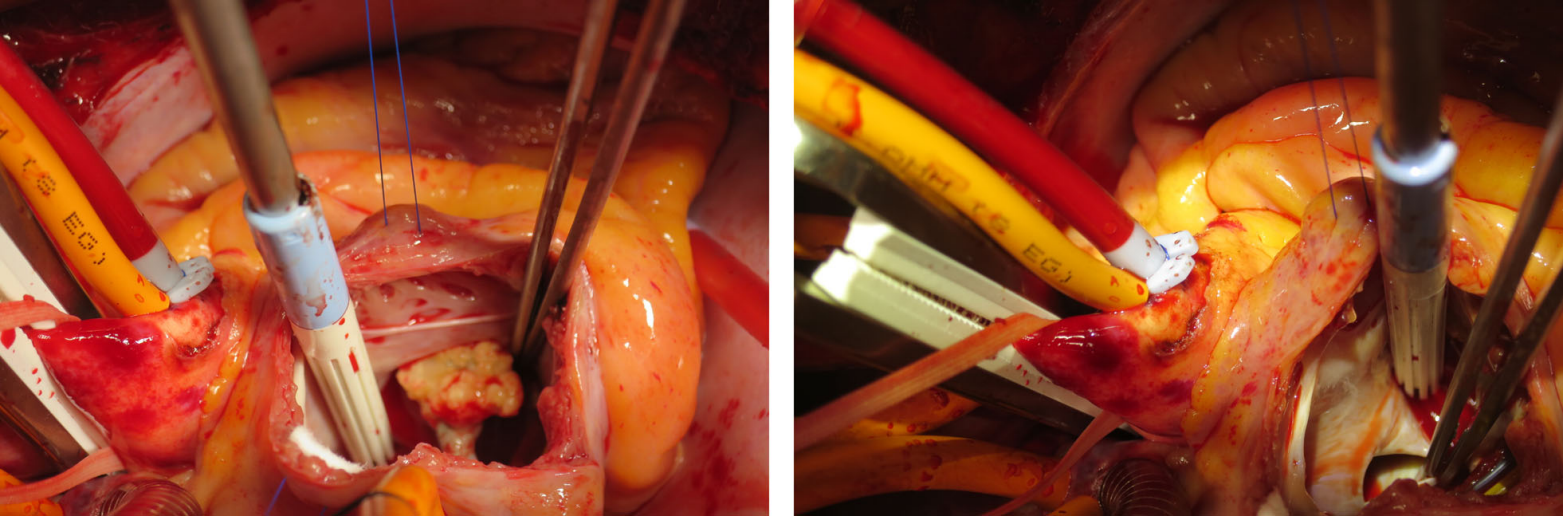
Closure of ASD by open heart surgery

The patient was admitted to our plastic and reconstructive surgery department for adjustment of the ICD position. She was found to have hypoxaemia and was accordingly referred to a cardiologist, who was unable to ascertain the cause of her hypoxaemia. Blood tests, including measurement of the D‐dimer concentration, showed no abnormalities. Lung function tests showed no obstructive or restrictive ventilatory disturbance: vital capacity, 2.81 L (which is 92% of predicted vital capacity); forced expiratory volume in 1 s, 84%; and low lung perfusion capacity (predicted %DLCO, 68.9 ml/min/mmHg/L). She was asymptomatic despite changes in her SpO_2_ with changes in posture (her SpO_2_ significantly decreased while standing). On room air, her SpO_2_ was 90% in the supine position but 80% when sitting. Cardiac ultrasound, chest radiography (Figure 1), and percutaneous echocardiography showed no abnormalities. Lung perfusion scintigraphy (Figure 2) and trans‐oesophageal echocardiography (Figure 3) to check for POS revealed a PFO and right‐to‐left shunt while sitting. Right heart catheterization showed no pulmonary hypertension. A review of the patient's previously obtained blood gas data showed mild to moderate hypoxaemia and hypocapnia with an abnormal alveolar–arterial difference in oxygen (Table 1). We diagnosed POS with an ASD, and cardiac repair surgery was performed (Figure 4). Although the patient exhibited no significant change in body weight between before and after the surgery, overnight SpO_2_ monitoring immediately postoperatively showed improvement in her hypoxaemia (Table 2). In particular, the CT90 value was remarkably improved, which was considered attributable to closure of the ASD. To our knowledge, this is the first evaluation of nocturnal SpO_2_ after ASD surgery in a patient with POS.

**TABLE 1:** Arterial blood gas findings before and after surgery (supine position)

	2 years prior to surgery (room air)	Admission for surgery (face mask 5 L/min)
SpO_2_ (%)	95	91
FiO_2_	0.21	0.4
pH	7.404	7.413
PaCO_2_ (mmHg)	29.9	34.6
PaO_2_ (mmHg)	67.9	77.0
HCO_3_^−^ (mmol/L)	18.3	19.4
BE (mmol/L)	−4.8	−3.3
A‐aDO2 (Torr)	31	

Abbreviations: A‐aDO_2_, alveolar–arterial oxygen difference; BE, base excess; FiO_2_, fraction of inspired oxygen; PaCO_2_, partial pressure of arterial carbon dioxide; PaO_2_, partial pressure of oxygen; SpO_2_, percutaneous arterial oxygen saturation.

**TABLE 2:** Portable overnight oximetry results before and after surgical closure of atrial septal defect

	Before surgery (room air)	After surgery (room air)
REI (event/hr)	33.5	17.0
3% ODI (event/hr)	32.7	16.7
Mean SpO_2_ (%)	94	98
Lowest SpO_2_ (%)	74	79
CT90 (%)	24.14	1.9

Abbreviations: CT90, cumulative percentage of sleep time with SpO_2_ of <90%; ODI, oxygen desaturation index; REI, respiratory event index; SpO_2_, percutaneous arterial oxygen saturation.

## DISCUSSION

POS is difficult to diagnose and is therefore often underdiagnosed.^1^ Although several case reports of POS have been published,^2^, ^3^, ^4^ none have addressed the pathophysiology of this condition in terms of its association with sleep. Exacerbation of hypoxaemia during anaesthesia has been reported,^5^ but how this is associated with disordered breathing during sleep remains unclear. POS is a condition associated with dyspnoea and hypoxaemia that improves in the supine position and worsens in the sitting position. In addition to cardiac shunt disease, the differential diagnoses of POS include pulmonary arterial varices and hepatopulmonary syndrome.^6^ In the present case, the respiratory disturbance index was particularly impaired during REM sleep. The worst hypoxaemia associated with respiratory events was characterized by an SpO_2_ of 80%, with the SpO_2_ averaging 93% in both REM sleep and NREM sleep. This was associated with respiratory events in the patient's polysomnography data. It seems likely that an intermittent right‐to‐left shunt is a trigger for severe OSA because hypopnea rather than obstruction is the main factor.

PFO and ASD can be caused by changes in the thoracic anatomy (e.g., as a result of chest surgery or aortic dilation). In our patient, the initial location of the ICD may have placed excessive pressure on the right atrium.^7^ OSA is associated with older age, male sex, and obesity, none of which applied in this case. Certain craniofacial characteristics are reportedly associated with the development of OSA, especially in East Asian patients.^8^ Our patient's small, short mandible was a risk factor for OSA. Additionally, persistent hypoxia exacerbates OSA, as in patients with overlap syndrome (coexistence of OSA and chronic obstructive pulmonary disease),^9^ and was a possible trigger in our patient. Trans‐oesophageal echocardiography and breath‐holding tests are helpful in achieving the correct diagnosis because the oxygen status changes with posture as a result of the anatomical characteristics and physiological changes in the thoracic cavity.^10^ A study of 188 patients showed that PFO and ASD were the most common causes of POS, and good results were achieved by percutaneous closure; however, many patients with congenital anomalies remain asymptomatic for years. Therefore, doctors should keep the diagnosis of POS in mind, especially when encountering patients with hypoxia of unknown cause or paroxysmal hypoxia. The diagnosis is simple and treatment is curative, achieving considerable improvements in patient autonomy and quality of life.^11^ Notably, with respect to the mechanism underlying POS, other possible causes include upper airway tumours (e.g., laryngeal cancer) and autonomic neuropathy.^12^, ^13^ Furthermore, cases of POS have recently been reported in patients with COVID‐19 pneumonia; this may be attributable to gravity‐induced redistribution of pulmonary blood flow.^14^, ^15^ CPAP treatment alleviates orthostatic hypoxia, suggesting extracardiac shunt‐related hypoxaemia.^16^ The present case is valuable in that OSA may have contributed to the manifestations of POS by the influence of nerve‐ and ventilation‐associated blood flow. Identification of severe hypoxaemia in a patient with OSA who is at low risk for hypoxaemia should prompt a search for rare causes of hypoxaemia, such as POS.

## AUTHOR CONTRIBUTIONS

Mamiko Hoshi, Kenichi Sugaya, Yusuke Jinno, Naho Furusho, Ryosuke Ozoe, Michio Kawamura, and Yutaka Kozu participated in the care of the patient. Mamiko Hoshi and Yutaka Kozu wrote the first draft of the manuscript. Hisato Hiranuma, Kazuo Chin, and Yasuhiro Gon helped to supervise the writing of the final version of the manuscript. All authors contributed to the conception of the work and revision of the manuscript, and all gave final approval of the version to be published.

## CONFLICT OF INTEREST STATEMENT

None declared.

## ETHICS STATEMENT

The authors declare that appropriate written informed consent was obtained for the publication of this manuscript and accompanying images.

## Data Availability

The data that support the findings of this study are available on request from the corresponding author. The data are not publicly available due to privacy or ethical restrictions.
